# Laparoscopic repair for perforated peptic ulcers with U-CLIP^®^

**DOI:** 10.1186/1749-7922-4-28

**Published:** 2009-07-29

**Authors:** Piero Guglielminotti, Roberto Bini, Diego Fontana, Renzo Leli

**Affiliations:** 1 Department of Surgery, San Giovanni Bosco Hospital, piazza Donatori di sangue 3, 10154, Torino, Italy

## Abstract

**Background:**

The literature established that, in patients without Boey's risk factors, laparoscopic repair of perforated peptic ulcers, compared to open repair, is associated to lower wound infection rate, less analgesic use, reduction in post operative pain, shorter hospital stay. Some of the main drawbacks are length of operative time and laparoscopic surgeon's experience in intracorporeal knotting.

We, for first, report our preliminary experience of perforated peptic ulcers' laparoscopic repair using Medtronic U-Clip^®^.

**Methods:**

From January 2008 to June 2008 we performed laparoscopic repair of perforated peptic ulcers using Medtronic U-Clip^® ^in 10 consecutive patients (6 men and 4 women, from 20 to 65 years-old of age). All the patients presented with iuxtapyloric perforated peptic ulcer, not greater than 10 mm, without signs of sepsis, free from major illnesses. The mini-invasive procedure was performed both by skilled and non-skilled laparoscopic surgeons under experts' surveillance. After it was recognized, perforation was sutured using U-Clip^® ^in a full-thickness manner.

**Results and Discussion:**

We reported no surgical complications in the peri-operative period. The clinical outcome and time needed to perform the intervention didn't change between skilled and non-skilled surgeons. The follow-up at 30 days was good.

**Conclusion:**

In our experience, the anastomotic device U-Clip^® ^simplifies laparoscopic repair of perforated peptic ulcer, avoiding the need to perform knots and making the procedure safe and easier.

## Background

Since the first laparoscopic repair of perforated peptic ulcer by Mouret in 1990 [1], mini-invasive technique has gained large popularity.

A research in electronic databases as Pub Med (meta-analysis, randomised control trial) and Cochrane review was conducted to identify the most relevant articles published between 1990 and 2008 regarding laparoscopic repair of perforated peptic ulcers.

In a meta analysis, Lau [2] identified that the post operative pain was lower than in open repair, and there was a significant reduction in wound infection, but reoperation rate was higher than open repair. Lau's conclusion was that laparoscopic repair was safe and effective for duodenal and juxtapyloric ulcers in patients without Boey's risk factors [3] (shock, major medical illnesses and longstanding perforation > 24 h).

Sanabria et al. [4] in a Cochrane database systematic review state that there were no statistically differences in septic abdominal complications between laparoscopic and open repair of perforated peptic ulcers.

Lunevicius et al. [5] in a systematic review confirm good results of laparoscopic repair in low risk patients in terms of lower analgesic use, shorter hospital stay, less wound infection, but define appropriate open repair in high risk patients and report in this case a shorter operation time than laparoscopic repair.

Moreover, Katkhouda et al. [6] report that laparoscopic repair for perforated duodenal ulcers is safe and maintains the benefits of minimally invasive approach (what means short hospital stay and less analgesic use), but still underline that laparoscopic repair is not beneficial in patients with shock and prolonged operation time than open repair.

Siu et al. [7] in a randomized controlled trial confirm the good results in terms of less post operative pain, less hospital stay, early return to normal daily activities, less chest infection, but introduce for the first time the concept that laparoscopic repair shortens surgical time procedure. These results are probably due to more restrictive indications for laparoscopic procedures. The Author's adopt conventional laparotomy in case of non-pyloric gastric ulcer, as well as in perforations larger than 10 mm and in presence of surgical technical difficulties.

Matsuda et al. [8] underline that laparoscopic ulcers repair requires surgeons with particular expertise in endoscopic surgery, but even a surgeon familiar with laparoscopic cholecystectomy can readily perform a laparoscopic approach after some practice.

Actually laparoscopic ulcers repair seems to be more effective compared to open treatment in case of juxtapyloric ulcers not greater than 10 mm in diameter, in absence of hemodynamic instability, hemorrhage, and inability to tolerate pneumoperitoneum [9].

Recently a new self-closing anastomotic device named U-Clip^® ^has been proposed in order to facilitate the anastomoses of vessels, grafts and other tubular structures during endoscopic and non-endoscopic surgery.

The U-Clip^® ^were used in the treatment of laparoscopic duodenal atresia [10].

We investigated the possibility to employ the U-Clip^® ^in the laparoscopic treatment of perforated peptic ulcers.

## Methods

Based on literature data we considered only patients with perforated ulcers in juxtapyloric position, not greater than 10 mm, in absence of signs of sepsis, without long-standing perforation and free from major medical illnesses. Surgery was performed by surgeons with different degree of laparoscopic experience. The diagnosis was obtained through orthostatic abdomen X-Ray and CT scan. No attempt was done to identify the ulcer location. If the perforation wasn't due to a juxtapyloric peptic ulcer or perforation larger than 10 mm, we changed strategy to laparotomy.

We used a thirty-degree optique and we put four trocars in the same position we usually adopt for laparoscopic cholecystectomy. Intravenous antibiotic therapy and inhibitor proton pump (omeprazole) were injected before insufflation. The abdomen was explored both to identify the site of perforation and to assess the severity of the peritonitis. Bacteriological samples were taken and sent immediately to the laboratory. After the perforation site was identified, we sutured it using 1 to 3 U-Clip^® ^stitches without omental patch. The U-Clip^® ^were passed directly at the edges of the perforation in a full-thickness manner and quickly closed by breaking the wire in the specific position. The abdomen was cleaned in each quadrant with about 5–6 liters of saline solution. We placed 1 or 2 drains (sub-hepatic and in the Douglas pouch). Trocars were removed under direct vision to look for abdominal wall bleeding. Postoperative antibiotic therapy was mantained for 5 days, in combination with long-time omeprazole administration. We suggested the execution of esophageal-gastro-duodenoscopy after 60 days.

## Results

From January 2008 to June 2008 we performed laparoscopic ulcer repair using U-Clip^® ^in 10 consecutive patients (6 men and 4 women, from 20 to 65 years-old of age) with juxtapyloric perforated ulcer, not greater than 10 mm, in absence of signs of sepsis. In our patients we reported no surgical complications. Feeding started after the return of peristalsis. The average operative time was approximately 65 minutes (± 25), mean hospital stay was 6 days.

Time needed to perform the intervention didn't change between skilled and non-skilled surgeons.

The follow-up at 30 days showed good conditions in all our patients (table 1. Results).

**Table 1:** Results

Mean age	42,5 ± 22.5
	
Sex	
Male	6
Female	4
Operative duration (minutes), Mean (SD)	65 ± 25
Postoperative hospital stay (days), Mean (SD)	6 ± 2
Food intake start (day post operative), Mean (SD)	4 ± 2
Follow up 30 days	10/10
Complications	None

## Discussion

Published data comparing laparoscopic and open repair for perforated peptic ulcers report lower post operative analgesic use, lower wound infection and mortality, fewer incisional hernias but longer operating time and higher reoperation rate.

Actually, operative techniques for laparoscopic ulcer repair include Graham-Steele patch repair, suture closure with an omental patch and simple closure without omental patch. The procedure is relatively simple but require the ability to perform an intracorporeal knot. The U-Clip^® ^device avoid the need to perform knots and make the procedure faster and easier.

The cost of U-Clip^®^, although higher than usual suture wires (1 U-Clip^® ^stich = 27,00 Euro; Polyglactin One stich = 3, 13 Euro), does not change in an important proportion the total cost of operation.

In our experience laparoscopic repair using U-Clip^® ^was performed also by not highly skilled surgeons under expert surgeons' surveillance, and the results in terms of duration of surgical procedure and clinical outcome were similar to those obtained by fully skilled laparoscopic surgeons.

## Conclusion

We verified the feasibility of an ulcer repair by mean of the new device U-Clip^®^. To our knowledge this is the first report of its use in this instance. We conclude that U-Clip^®^, avoiding intracorporeal knots, simplify the laparoscopic procedure.

No significative costs are added to laparoscopic procedure using U-Clip^®^.

Further controlled-randomized trials will be necessary to determine whether U-Clip^® ^compares favourably with the classical intracorporeal knotting technique in the laparoscopic repair of perforated peptic ulcers in the majority of patients.

## Competing interests

The authors declare that they have no competing interests.

## Authors' contributions

GP: Conceived the study, and participated in its design. BR: Co-conceived the study and participated in its coordination. FD: Acquisition and interpretation of data. LR: Revision of manuscript and participate in its design. All Authors read and approved the final manuscript.
